# Frequency of Interstitial Lung Disease in Rheumatoid Arthritis Patients: A Hospital-Based Study

**DOI:** 10.7759/cureus.30145

**Published:** 2022-10-10

**Authors:** Zafeer-ul-hassan Iqbal, Jawad A Khan, Muhammad Mujeeb Hassan, Rafay Zaidi, Azka A Mirza, Moeena Malik, Hira Ashraf, Maham Javaid, Adil Mahmood, Khurram Zohaib

**Affiliations:** 1 Department of Internal Medicine, District Headquarter Hospital (DHQ), Rawalpindi, PAK; 2 Department of Internal Medicine, Saidu Teaching Hospital-Saidu Medical College, Swat, PAK; 3 Department of Internal Medicine, Birmingham Heartlands Hospital, Birmingham, GBR; 4 Department of Internal Medicine, Rawalpindi Medical University, Rawalpindi, PAK; 5 Department of Obstetrics and Gynecology, District Headquarter Hospital (DHQ), Rawalpindi, PAK; 6 Department of Internal Medicine, Allama Iqbal Medical College, Lahore, PAK; 7 Department of Internal Medicine, Shifa International Hospital Islamabad, Islamabad, PAK; 8 Department of Internal Medicine, Benazir Bhutto Hospital, Rawalpindi, PAK

**Keywords:** complication of rheumatoid arthritis, interstitial lung, pulmonary involvement, extra-articular manifestations, : rheumatoid arthritis

## Abstract

Introduction: Among various extra-articular manifestations of rheumatoid arthritis (RA), interstitial lung disease (ILD) is the most frequent and concerning manifestation. The reported frequency of RA-associated ILD (RA-ILD) varies in the literature. The objective of the present study was to determine the frequency of ILD in RA patients at a teaching hospital in Rawalpindi.

Methods: 175 male and female patients between 18-70 years were enrolled in the study from January 21, 2022, till July 24, 2022. Patients diagnosed with RA were screened for the concomitant presence of ILD (increased pulmonary markings on chest X-rays and total lung capacity ≤ 80%, predicted on pulmonary function tests). In addition, the frequency of RA-ILD was noted and compared across various subgroups of patients based on age, gender, and disease duration.

Results: The mean age of the patients was 45.3 ± 11.5 years. The male-to-female ratio was 1:3.1. The mean disease duration was 6.2 ± 3.5 years at the time of presentation. A total of 118 (67.4%) patients were diagnosed with RA-ILD. The frequency of RA-ILD was significantly higher among patients with a prolonged duration of disease, < 5 years vs. ≥ 5 years (59.1% vs. 75.9%; p-value=0.018). Among 118 patients with RA-ILD, usual interstitial pneumonia (UIP) was the most frequent pattern and was noted in 74 (62.7%) patients, followed by nonspecific interstitial pneumonitis (NSIP), which was noted in 44 (37.3%) patients. When compared, there was no statistically significant difference in the frequency of high-resolution CT (HRCT) pattern of RA-ILD across various subgroups of patients based on age (p-value=0.969), gender (p-value=0.934), and duration of disease (p-value=0.881).

Conclusion: In the present study, a substantial proportion of RA patients suffered RA-ILD, which warrants routine screening of these patients for undiagnosed pulmonary involvement so that timely identification and anticipated management may improve the outcome of such cases in future clinical practice.

## Introduction

Interstitial lung disease (ILD) is the most common extra-articular manifestation of rheumatoid arthritis (RA) in the lung and contributes to morbidity and mortality in patients with RA. ILDs are diseases in which inflammation and fibrosis diffusely impact the pulmonary interstitium and parenchyma. ILDs include a variety of subsets, such as idiopathic interstitial pneumonia (IIP), collagen vascular disease-related ILD, and chronic hypersensitivity pneumonitis [1]. Although ILDs can show chronic or sub-acute progression, they sometimes show acute exacerbation leading to respiratory failure, which is associated with significant mortality [2,3].

RA-associated ILD (RA-ILD) may be a consequence of the chronic immune activation and inflammation that occurs in RA and subsequently promotes aberrant fibro proliferation or can be due to drug-related or infectious precipitants [4]. RA-ILD contributes significantly to a decrease in the quality of life, progressive chronic disability, high utilization of healthcare resources, and mortality rate, with a mean survival of under three years [5].

It has been estimated that nearly 50% of RA patients will develop some form of respiratory abnormality during their lifetime [6]. RA mainly affects the lining of the synovial joints; however, its sequelae can result in progressive disability, premature death, and socioeconomic burdens. The clinical presentations of symmetrical joint involvement include arthralgia, swelling, redness, and even restricting the range of motion. Therefore, early diagnosis is essential for desirable outcomes (i.e., reduced joint destruction, less radiologic progression, no functional disability, and disease-modifying anti-rheumatic drugs-free remission) and cost-effectiveness. The optimal therapeutic window is the first 12 weeks after early symptoms occur [7,8].

Bongartz et al. (2010) reported the frequency of ILD to be 7.9% in RA patients; the lymphocytic interstitial pneumonia (LIP) pattern was present in 19.6%, and the nonspecific interstitial pneumonitis (NSIP) pattern was present in 2.2% among ILD patients [9]. Perez et al. (2015) reported this frequency to be 34% among RA patients in Mexico, and NSIP was the most prevalent pattern on high-resolution CT (HRCT) scans (29%). The usual interstitial pneumonia (UIP) pattern was observed in 13% of the patients [10]. A lower frequency of 3.6% has been reported by Richman et al. (2013) in Hispanic and Asian patients with RA [11]. A much higher frequency of 71.6% has been reported by Fadda et al. (2018) among Egyptian patients, and ILD patterns were UIP 62% & NSIP 27% [12].

In light of the above, ILD is a frequent extra-articular manifestation of RA. However, the frequency of ILD-RA varied immensely in different studies. Therefore, there is a controversy about the frequency of ILD in the literature, from as low as 3.6% to as high as 71.6% [11,12]. A possible explanation for this conflict can be a variation in the ILD definition, and RA severity in the population studied. However, the existing evidence is limited. Therefore, to confirm the extent of this variation, this study was re-conducted in the Asian population.

## Materials and methods

A total of 175 patients were enrolled in the study from January 21, 2022, till July 24, 2022, at the outdoor clinic of the Department of Medicine, District Headquarters Hospital, Rawalpindi, Pakistan. Patients were selected by non-probability, consecutive sampling.

Written informed consent and a detailed history were taken from every patient before enrolment. Patients were screened for ILD. Chest radiographs, HRCT, and pulmonary function tests (PFT) were performed for each patient. PFTs and radiographs of the thorax were performed according to the standard protocol. Serial slices were taken from the chest, each 1 mm in width and 10 mm apart. chest radiograph was performed at the same time as the PFTs. Two consultant radiologists interpreted PFTS and X-rays.

ILD was suspected in RA patients presenting with a history of chronic cough (more than three months). ILD was diagnosed if the X-ray showed increased pulmonary markings and restrictive pattern obtained on PFT (TLC ≤80% predicted). In addition, the patient's demographic details, disease duration, and RA-induced lung damage were also noted and recorded in an attached proforma. All the PFTs and X-rays were conducted at the same (hospital) lab to eliminate bias. Confounding variables were controlled by exclusion.

The inclusion criteria included adult patients, male or female ≥18 years of age at the time of enrolment, presenting with RA. The exclusion criteria included patients with diabetes mellitus (fasting blood sugar ≥110mg/dl), pre-existing liver disease (serum bilirubin ≥1.0 mg/dl), chronic kidney disease (serum urea ≥40 mg/dl and creatinine ≥1.2 mg/dl), patients with post-infectious fibrosis or bronchiectasis, and alcoholic patients (≥100 ml of alcohol/day).

Statistical analysis 

Data were analyzed using IBM SPSS Statistics for Windows, Version 25.0 (Released 2017; IBM Corp., Armonk, New York, United States). Numerical variables such as age and duration of disease were presented as mean ± standard deviation. The categorical variables, i.e., gender, RA-ILD, and HRCT pattern (NSIP, UIP), were presented using frequency and percentage. Data was stratified for age, gender, and duration of disease. Post-stratification, the chi-square test was applied, and a p-value of ≤0.05 was considered significant.

Ethical disclosure

This study was approved by the ethics committee of District Headquarters Hospital, Rawalpindi, Pakistan, and was conducted in accordance with the Declaration of Helsinki. Written informed consent was taken from all the patients before the start of the trial.

## Results

The age of the patients ranged from 18 to 70 years, with a mean of 45.3 ± 11.5 years. Majority (n=104, 59.4%) of the patients were aged <45 years. There were 43 (24.6%) males and 132 (75.4%) females with a male-to-female ratio of 1:3.1. The disease duration in our study participants ranged from one to 12 years with a mean of 6.2 ± 3.5 years, as shown in Table 1. A total of 118 (67.4%) patients with RA had RA-ILD, as shown in Table 2. When compared, there was no statistically significant difference in the frequency of RA-ILD across various subgroups of patients based on age (p-value=0.967) and gender (p-value=0.998). However, it was significantly higher among patients with a prolonged duration of disease, <5 years vs. ≥5 years (59.1% vs. 75.9%; p-value=0.018), as shown in Table 3. Among 118 patients with RA-ILD, UIP was the most frequent pattern and was noted in 74 (62.7%) patients, followed by NSIP, which was noted in 44 (37.3%) patients, as shown in Table 4. When compared, there was no statistically significant difference in the frequency of HRCT pattern of RA-ILD across various subgroups of patients based on age (p-value=0.969), gender (p-value=0.934), and duration of disease (p-value=0.881) as shown in Tables 5, 6, 7, respectively.

**Table 1:** Demographic characteristics of patients with rheumatoid arthritis

Characteristics	Participants (n=175)
Age (years)	45.3±11.5
<45 years	104 (59.4%)
≥45 years	71 (40.6%)
Gender	
Male	43 (24.6%)
Female	132 (75.4%)
Duration of Disease (years)	6.2±3.5
<5 years	88 (50.3%)
≥5 years	87 (49.7%)

**Table 2:** Frequency of rheumatoid arthritis-associated interstitial lung disease (RA-ILD) in study cohort (n=175)

Rheumatoid Arthritis-Associated Interstitial Lung Disease	Frequency (n)	Percent (%)
Yes	118	67.4 %
No	57	32.6 %
Total	175	100.0 %

**Table 3:** Frequency of rheumatoid arthritis-associated interstitial lung disease (RA-ILD) across various subgroups of rheumatoid patients (n=175)

Subgroups	n	RA-ILD n (%)	P-value
Age			
<45 years	104	70 (67.3%)	0.967
≥45 years	71	48 (67.6%)	
Gender			
Male	43	29 (67.4%)	0.998
Female	132	89 (67.4%)	
Duration of Disease			
<5 years	88	52 (59.1%)	0.018*
≥5 years	87	66 (75.9%)	

**Table 4:** Frequency of various high-resolution CT (HRCT) patterns among patients with rheumatoid arthritis-associated interstitial lung disease (RA-ILD) (n=118)

HRCT Pattern of RA-ILD	Frequency (n)	Percent (%)
Usual Interstitial Pneumonia (UIP)	74	62.7 %
Nonspecific Interstitial Pneumonitis (NSIP)	44	37.3 %
Total	118	100.0 %

**Table 5:** Frequency of various high-resolution CT (HRCT) patterns in patients with rheumatoid arthritis-associated interstitial lung disease (RA-ILD) by age (n=118) UIP = usual interstitial pneumonia; NSIP = nonspecific interstitial pneumonitis

Age			HRCT Pattern		Total	P-value
			UIP (n=74)	NSIP (n=44)		
	<45 years (n=70)		44	26	70	0.969
			62.9%	37.1%	100.0%	
	≥45 years (n=48)		30	18	48	
			62.5%	37.5%	100.0%	
Total			74	44	118	
	62.7%	37.3%	100.0%	

**Table 6:** Frequency of various high-resolution CT (HRCT) patterns in patients with rheumatoid arthritis-associated interstitial lung disease (RA-ILD) by gender (n=118) UIP = usual interstitial pneumonia; NSIP = nonspecific interstitial pneumonitis

Gender			HRCT Pattern		Total	P-value
			UIP (n=74)	NSIP (n=44)		
	Male (n=29)		18	11	29	0.934
			62.1%	37.9%	100.0%	
	Female (n=89)		56	33	89	
			62.9%	37.1%	100.0%	
Total			74	44	118	
	62.7%	37.3%	100.0%	

**Table 7:** Frequency of various high-resolution CT (HRCT) patterns in patients with rheumatoid arthritis-associated interstitial lung disease (RA-ILD) by the duration of disease (n=118) UIP = usual interstitial pneumonia; NSIP = nonspecific interstitial pneumonitis

Duration of Disease			HRCT Pattern		Total	P-value
			UIP (n=74)	NSIP (n=44)		
	<5 years (n=52)		33	19	52	0.881
			63.5%	36.5%	100.0%	
	≥5 years (n=66)		41	25	66	
			62.1%	37.9%	100.0%	
Total			74	44	118	
	62.7%	37.3%	100.0%	

## Discussion

RA, characterized by inflammatory joint destruction, is the most common connective tissue disease. The global prevalence of RA is 0.24% (or 16 million people), and it ranks as the 42nd highest contributor to global disability [13]. RA usually presents as pain in one or more joints over several weeks to months and morning stiﬀness lasting more than one hour, which usually improves with exercise [13,14]. When not effectively treated at an early stage, the chronic inﬂammatory process caused by RA results in joint destruction, leading to disability, decreased functional status, unemployment, and reduced quality of life and expectancy [13-15]. The expected survival of RA patients is expected to be reduced by 3-10 years compared with that of the general population. The decreased life expectancy is associated with diverse systemic manifestations and complications related to the treatment. Systemic presentations and complications of RA include pulmonary, cardiovascular, neurological, and musculoskeletal involvements; glucocorticoid-induced osteoporosis; and infection. These complications have significant impacts on the disease outcome and occur in approximately 40% of patients [13].

In RA, the type of lung disease and its manifestations vary and can affect any lung compartment, including the lung parenchyma, pleura, airways, and vasculature [4,5]. RA-ILD is the most common form of lung parenchymal involvement in RA [5]. The reported frequency of RA-ILD varies in the published literature owing to population differences in the studies [9-12]; furthermore, there is data in literature based on Pakistan's local population; this necessitated the present study to get an insight into the magnitude of the problem and provide baseline statistical data for further research in this regard.

The objective of the present study was to determine the frequency of ILD in RA patients at a teaching hospital in Rawalpindi. In the present study, the mean age of the patients with RA was 45.3 ± 11.5 years. Our results align with that of Wagan et al. (2016), who reported a similar mean age of 45.1±9.5 years among patients with RA at Sheikh Zayed Federal Post Graduate Medical Institute, Lahore, Pakistan [16]. Imran et al. (2015) observed a comparable mean age of 43.5 ± 11.9 years among such patients presenting at Fatima Memorial Hospital, Lahore, Pakistan [17]. In another study conducted at the same institute, Ahmad et al. reported it to be 46 ± 12.6 years in line with the present study [18]. In a similar study involving Indian patients with RA, Dev et al. observed a mean age of 45 ± 2.6 years.

We observed female predominance with a male-to-female ratio of 1:3.1. Our observation is in line with that of Wagan et al. They evaluated RA presenting at Central Park Medical College Hospital, Lahore, Pakistan, and observed a similar female predominance with a male-to-female ratio of 1:3.1 [19]. In addition, similar female predominance (M:F; 1:3.0) has also been reported by Zafar et al. (2016) among RA patients presenting at Shaikh Zayed Hospital, Lahore, Pakistan [20]. RA was seen more frequently in middle-aged females. We observed that 67.4% of patients with RA had RA-ILD. Among patients with RA-ILD, UIP was the most frequent pattern and was noted in 74 (62.7%) patients, followed by NSIP, which was noted in 44 (37.3%) patients.

In a recent study of over 645 American patients diagnosed with RA, Samhouri et al. observed a comparable frequency of RA-ILD and reported it to be 67.0% [21]. Our observation is also in line with an Egyptian study where Samy et al. evaluated lung function among 160 RA patients and reported RA-ILD in 63.8% of patients. They also reported similar distribution of UIP (60.7%) and NSIP (39.3%) HRCT patterns among these patients [22]. In addition, a comparable frequency of 61.0% has been reported by Wang et al. (2015) in Chinese rheumatoid patients [7].

Some patients with RA-ILD may experience sudden clinical deterioration due to acute exacerbations of ILD (ILD-AE). The clinical presentation is similar to the worsening respiratory status observed in idiopathic pulmonary fibrosis (IPF). In a study by Shinji et al., UIP was the most reported ILD pattern associated with ILD-AE. Multiple logistic regression analysis revealed a significant correlation of UIP with ILD-AE (p value= 0.038) [23]. However, data on the prognosis of patients with ILD-AE is yet to be reported on a larger scale.

One of the study limitations that should be addressed is the use of methotrexate (MTX) by our study participants. MTX, the primary drug for the treatment of RA, has been associated with lung injury and, in particular, with MTX-related pneumonitis. MTX use can result in pulmonary fibrosis in patients and can present with nonspecific symptoms like progressive dry or productive cough and dyspnea, with or without fever. These symptoms are also associated with ILD. In our study, patients diagnosed with RA and with signs and symptoms of ILD were selected. Due to recall bias, the duration of use of MTX by these patients could not be ascertained, and MTX's role in aiding in pulmonary fibrosis could not be assessed in our study participants. In a meta-analysis conducted by Conway et al., the use of MTX in RA patients was associated with a small but significant increase in the risk of lung disease compared with other disease-modifying antirheumatic drugs and biologic agents [24].

The present study is the first of its kind based on the local population and adds to the limited published international research evidence on the topic. In this study, we observed that a substantial proportion of RA patients suffered RA-ILD, which warrants routine screening for undiagnosed pulmonary involvement so that timely identification and anticipated management may improve the outcome in future clinical practice. We also observed that the frequency of RA-ILD increased significantly with increasing duration of rheumatoid arthritis; <5 years vs. ≥5 years (59.1% vs. 75.9%; p-value=0.018), suggesting a potential role of disease duration in the risk stratification and management planning of such cases.

The strengths of the present study were its large sample size of 175 cases and strict exclusion criteria. We also stratified the data to address various effect modifiers like the patient's age, gender, and disease duration. An extreme limitation of the present study was that we did not consider the RA management's effect on the clinical control of ILD and improvement in pulmonary function. Such a study is imperative and is highly recommended in future clinical research.

## Conclusions

In the present study, a substantial proportion of RA patients suffered RA-ILD, which warrants routine screening for undiagnosed pulmonary involvement so that timely identification and anticipated management may improve the outcome of such cases in future clinical practice. The impact of ILD on patient survival provides evidence that evolving more promising strategies for treating ILD could significantly reduce the excess mortality of individuals with RA.
